# Perineal endometriosis: a rare case of a unique sizeable nodule

**DOI:** 10.11604/pamj.2021.38.47.27737

**Published:** 2021-01-18

**Authors:** Hana Hakim, Sawssan Ben Halima, Ahmed Zouari, Dora Trabelsi, Mohamed Derbel, Kais Chaabane, Sahbi Kebaili

**Affiliations:** 1 University of Medicine of Sfax, Department of Gynecology and Obstetrics, Hedi Chaker Hospital, 3029, Sfax, Tunisia

**Keywords:** Perineal, endometriosis, surgical, sizeable, case report

## Abstract

Endometriosis is the presence of endometrial tissue in abnormal locations outside the uterine cavity. These locations are usually the ovaries, the peritoneum, and the uterine ligaments. Less frequently, the endometrial tissue can affect the perineum especially after surgical procedures or obstetric lesions. In this case report, we describe the case of a patient suffering from pain and swelling, with a sizeable nodule in an old episiotomy scar. Clinical examination, ultrasonography (USG) and magnetic resonance imaging (MRI) helped evoke the diagnosis of perineal endometriosis, and there were no signs of other endometriosis locations. Hormonal treatment was provided at first, but there was no clinical improvement after three months, so the treatment had to be surgical. Histopathological examination of the endometriotic mass confirmed the diagnosis. There were no immediate postoperative complications, and no clinical symptoms or recurrence signs six months and one year after.

## Introduction

Endometriosis is defined by the presence of endometrial tissue outside the uterine cavity [1]. It is a fairly common disease affecting women of reproductive age, and its prevalence varies between 10% to 25% [2]. Perineal endometriosis, however, is a relatively rare occurrence that only affects between 0.3% and 1% of women [3]. Schikele was the first to address the issue in 1923, but since then, there still are very few studies in the literature worldwide covering this disease. Perineal endometriosis is defined by the presence of endometrial cells in the perineum [4]. It usually affects women who have an old episiotomy scar, an obstetric tear or even a caesarean scar in their medical history. The pathogenesis of perineal endometriosis is still not clearly established despite the many theories surrounding the matter. The most approved theory is direct implantation of the endometrial cells on scars during the obstetric trauma [4]. Complementary exams are usually prescribed, but the diagnosis can only be confirmed after histopathological examination of the material [3]. In this study, we will describe a new case of episiotomy scar endometriosis in order to outline the specifications of this affection, mainly diagnostic means, therapeutic ways and prognosis of this rare affection.

## Patient and observation

**Figure 1:**
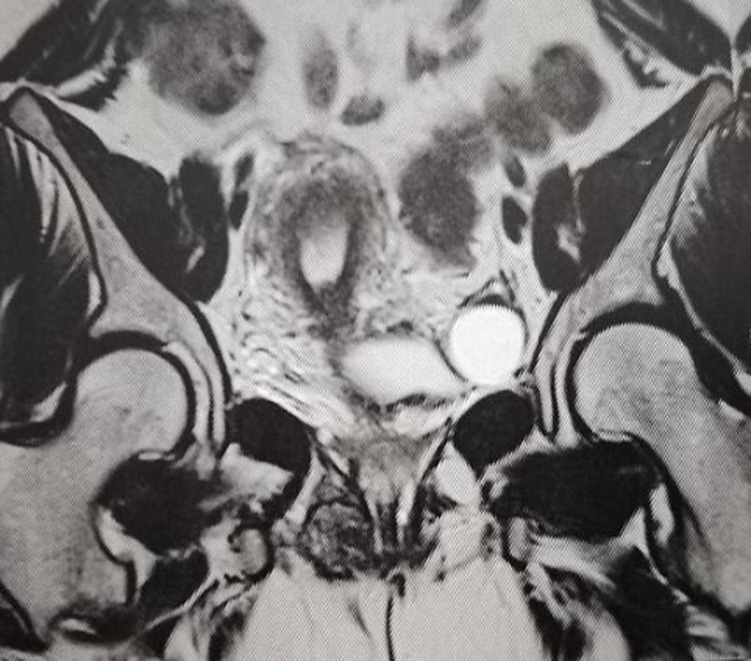
T2-weighted MRI image, coronal plane through pelvis showing the endometriotic nodule (arrow)

In this study, we present the case of a 34-year-old woman who consulted our emergency room after the development of a painful mass at the episiotomy site, paired with perineal pain and pruritus. This patient had two previous vaginal deliveries; the first one in 2016 and the second in January 2018. Both newborn babies weighed under 4000g, and the deliveries occurred without complications, except a right mediolateral episiotomy in both cases. The symptoms, at first some perineal tenderness that evolved into mild pain, started approximatively one year after the second delivery. The patient reported that her symptoms usually got worse on the menstrual period and the few days prior to that. Upon inspection, the perineum appeared normal. Vaginal examination revealed a 3cm x 2cm mass on the old episiotomy scar. The pelvic ultrasonography (USG) showed no signs of endometriosis on the uterus or the ovaries. A pelvic magnetic resonance imaging was performed, and showed a 45 x 22 x 26cm heterogeneous mass with hemorrhagic signal developed in the right ischiorectal fossa (Figure 1). This sizeable mass was adjacent to the external anal sphincter while the internal anal sphincter was preserved. There were no signs of other deep endometriosis locations on the magnetic resonance imaging.

**Figure 2:**
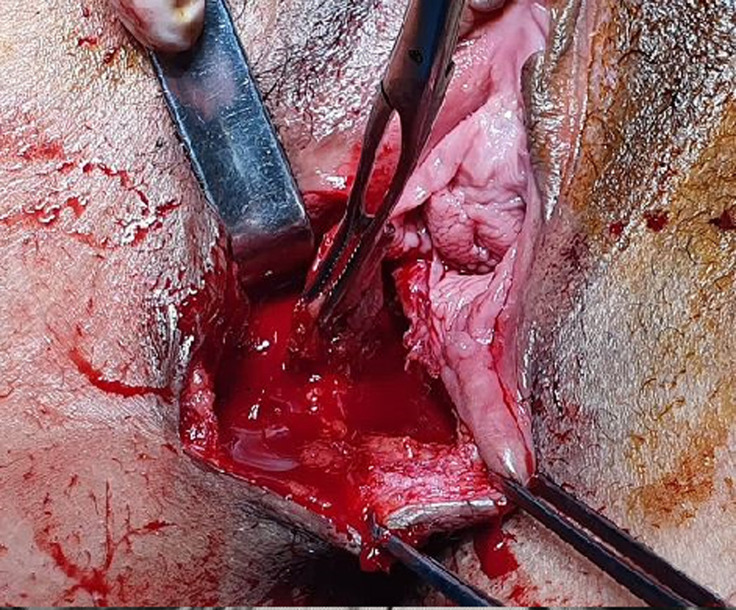
deep endometriotic nodule

**Figure 3:**
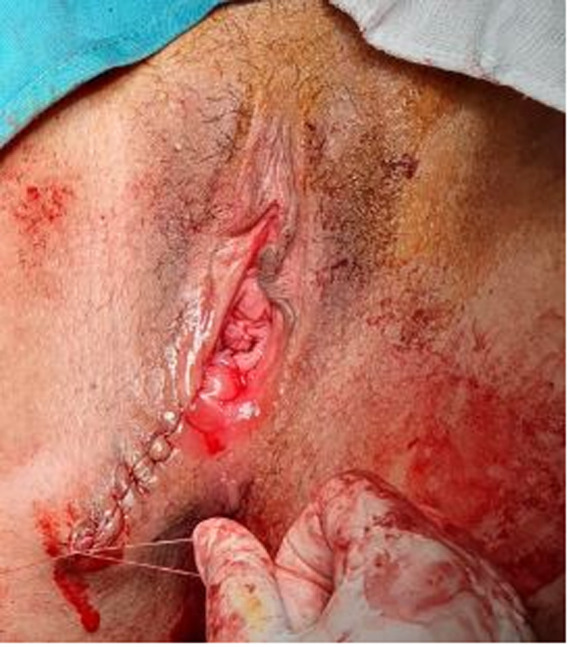
postoperative result

**Figure 4:**
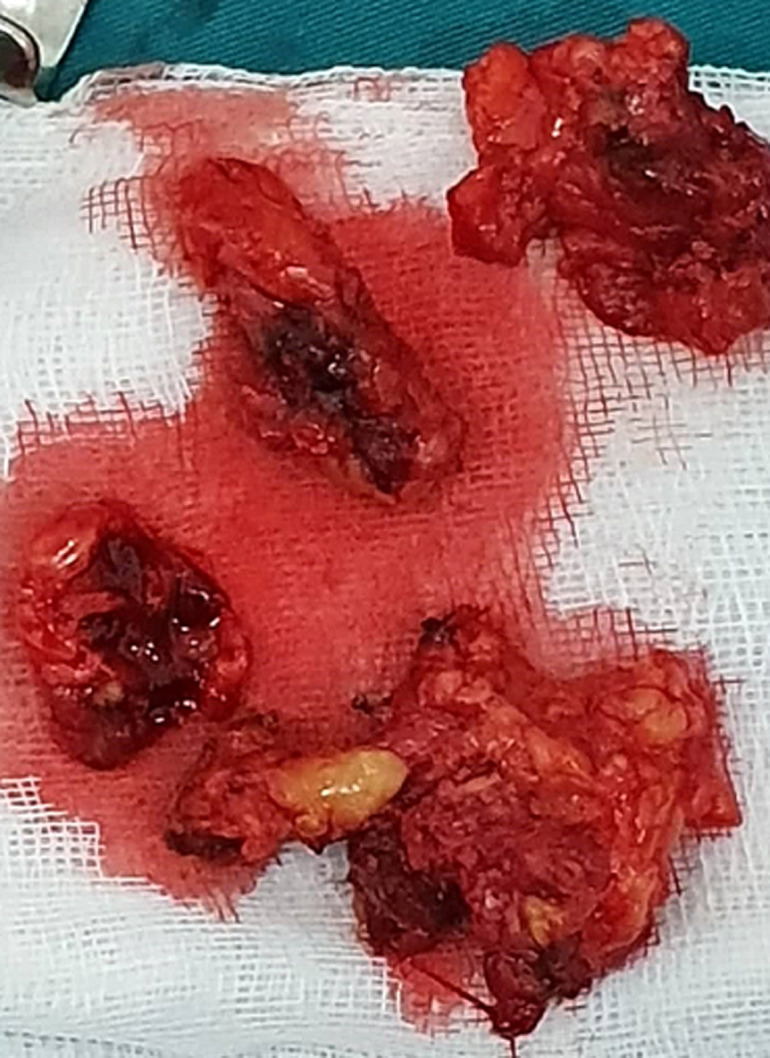
endometriotic masses

**Figure 5:**
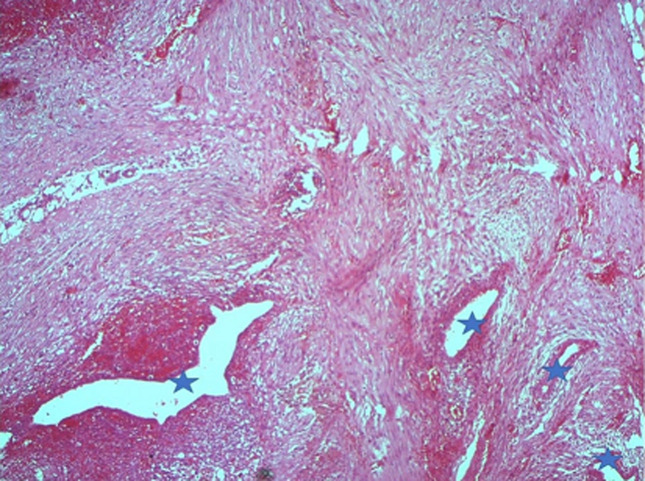
fibrous tissue showing endometrial type glands with stroma (star)

The diagnosis of perineal endometriosis was suspected, and hormonal therapy was started. However, there was no clinical improvement after three months of dienogest, as the nodule size didn´t decrease and the patient was still in pain. A complete surgical excision under general anesthesia was decided. The gynecologist started by making an incision on the episiotomy scar. Electrosection was used next in order to minimize bleeding and to excise the mass, as well as a significant margin of healthy tissue (Figure 2). The accidental section of the mass showed that the substance composing the mass was a chocolate brown liquid, a clear indication of endometriosis. The postoperative result is shown in Figure 3. The histopathologic examination found 5 fragments, the biggest one measuring 3 x 3 x 1cm (Figure 4). The fragments contained fibroadipose tissue, glandular structures and endometrial epithelium, which are the usual components of endometriotic tissue. There was no evidence of malignancy. The diagnosis of endometriosis was confirmed (Figure 5). There were no immediate postoperative complications, and the patient was discharged on the fourth postoperative day. Follow-ups were conducted six months and one year after surgery. The patient was asymptomatic, and had no signs of recurrence whatsoever.

## Discussion

Perineal endometriosis is a rare disease affecting women of reproductive age. The signs associated to this condition are variable and nonspecific, and the clinical diagnosis is usually difficult. In addition, the pathogenesis of this affection is still unclear. However, the most agreed upon theory is the autologous implantation of endometrial cells in an open episiotomy wound (during vaginal delivery) [1]. Those cells would divide, form a mass and respond to cyclic hormonal stimulations. The diagnosis should be evoked when a young woman of reproductive age feels pain or discovers a lump or a mass in her anoperineal region. Zhu *et al*. conducted a retrospective study of 36 cases of perineal endometriosis, and concluded that three criteria were essential to the diagnosis. If these three criteria were all present, the predictive value of perineal endometriosis was 100% [5]. These criteria are: past episiotomy scar or perineal tear, of gynecological origin, in the patient´s history; a hard perineal tumefaction, usually painful to the touch, bluish or even brownish; cycling pain and swelling of the perineal tumefaction during the menstrual cycle.

Perineal examination should always be performed when a patient complains of chronic perineal pains, especially if she has an episiotomy scar. This simple examination usually shows the perineal nodule, and the gynecologist has to look for similar nodules in the anorectal region and on the rectovaginal septum. Perineal endometriosis should not be confused with anoperineal abscess, which is a localized, fluctuating, and recurring perineal mass. Anal melanoma should also be evoked despite its rareness [6]. The two imaging exams that are usually performed to help confirm the diagnosis of endometriosis are ultrasonography and magnetic resonance imaging [7]. Sonographic findings are not specific, and a heterogeneous hypoechoic lesion with internal echoes is the most common feature in perineal endometriosis [5]. Magnetic resonance imaging is now considered the best imaging technique to assess endometriosis [7]. It can be useful in analyzing pelvic and extra pelvic locations and detecting other implantation sites, even distant ones, but its input is much more significant with deep pelvic endometriosis [8]. Magnetic resonance imaging is also essential to assess extension to the anal sphincter [9]. The presence of T1 and T2 hyper-intensity and the absence of fat saturation favor the diagnosis of endometriosis [8].

Treatment of perineal endometriosis is exclusively surgical [2]. A large local excision is performed on the nodule, taking the whole mass as well as a 1cm margin of healthy tissue [9]. If not treated early, the endometriotic lesions may attain the anal sphincter and progress into fecal incontinence. More importantly, malignant transformation into adenocarcinomas or sarcomas was reported in 0.3% of cases in the literature [10]. Incomplete excision of the mass can lead to recurrence, usually within a year [3]. Furthermore, the histopathological examination of the lesion is necessary to obtain a definite diagnosis. The most common cytology findings are endometrial glandular epithelium, ovoid stromal cells, and macrophages in a hemorrhagic background [3].

## Conclusion

Perineal endometriosis should always be evoked whenever a woman of reproductive age has cyclic pains and swelling in the site of an episiotomy scar. Clinical examination is usually enough for the diagnosis, but can be associated to ultrasound and MRI in case of doubt. However, the diagnosis can only be certain when histopathological examination is performed on the mass. The treatment is exclusively surgical (a large excision of the endometriotic mass), and the evolution is usually favorable. The clinician should always keep in mind that perineal endometriosis could be associated to deeper lesions of the same nature, especially in the ovaries.
